# Development and Evaluation of Thermosensitive Hydrogels with Binary Mixture of *Scutellariae baicalensis radix* Extract and Chitosan for Periodontal Diseases Treatment

**DOI:** 10.3390/ijms222111319

**Published:** 2021-10-20

**Authors:** Justyna Chanaj-Kaczmarek, Tomasz Osmałek, Emilia Szymańska, Katarzyna Winnicka, Tomasz M. Karpiński, Magdalena Dyba, Marta Bekalarska-Dębek, Judyta Cielecka-Piontek

**Affiliations:** 1 Department of Pharmacognosy, Faculty of Pharmacy, Poznan University of Medical Sciences, 4 Swiecickiego Street, 61-781 Poznan, Poland; justyna.chanaj-kaczmarek@ump.edu.pl; 2 Department of Pharmaceutical Technology, Faculty of Pharmacy, Poznan University of Medical Sciences, 6 Grunwaldzka Street, 60-780 Poznan, Poland; tosmalek@ump.edu.pl; 3 Department of Pharmaceutical Technology, Faculty of Pharmacy, Medical University of Bialystok, 2c Mickiewicza Street, 15-222 Białystok, Poland; emilia.szymanska@umb.edu.pl (E.S.); kwin@umb.edu.pl (K.W.); 4 Chair and Department of Medical Microbiology, Medical Faculty, Poznan University of Medical Sciences, 3 Wieniawskiego Street, 61-712 Poznan, Poland; tkarpin@ump.edu.pl; 5 Department of Prosthodontics and Dental Technology, Faculty of Dentistry, Poznan University of Medical Sciences, 70 Bukowska Street, 60-812 Poznan, Poland; magdalena.nowak@ump.edu.pl; 6 Department of Conservative Dentistry and Periodontology, Faculty of Dentistry, Poznan University of Medical Sciences, 70 Bukowska Street, 60-812 Poznan, Poland; m.bekalarskadebek@ump.edu.pl

**Keywords:** *Scutellaria baicalensis*, chitosan, periodontal diseases, dissolution studies, ex vivo mucoadhesion, permeability

## Abstract

*Scutellaria baicalensis* root displays anti-inflammatory and antibacterial properties due to the presence of flavonoids, particularly baicalin, baicalein, and wogonin. Our work aimed at developing thermosensitive hydrogels containing a binary mixture of *S. baicalensis radix* lyophilized extract and chitosan as a novel approach for periodontal diseases treatment. Two types of chitosan were employed in preliminary studies on binary mixtures with *S. baicalensis radix* lyophilized extract standardized for baicalin, baicalein, and wogonin. Thermosensitive hydrogels were prepared of poloxamer 407, alginate sodium, and cellulose derivatives and evaluated in terms of rheological and mucoadhesive behavior. The presence of chitosan altered the release profile of active compounds but did not affect their in vitro permeation behavior in PAMPA assay. The synergistic effects of *S. baicalensis radix* lyophilized extract and chitosan toward ferrous ion-chelating activity, inhibition of hyaluronidase, and pathogen growth were observed. The thermosensitive gelling system showed shear-thinning properties, gelation temperature between 25 and 27 °C, and favorable mucoadhesiveness in contact with porcine buccal mucosa, which was enhanced in the presence of binary mixture of *S. baicalensis radix* extract and chitosan. The release tests showed that baicalin and baicalein were liberated in a prolonged manner with a fast onset from hydrogel formulations.

## 1. Introduction

Periodontitis is a chronic immuno-inflammatory disease that causes the destruction of periodontal tissues and the alveolar bone supporting the teeth [1]. Periodontitis affects more than 10% of the adult population, making it the 11th most prevalent disease globally, and it is associated with systemic diseases such as diabetes, cardiovascular disease, rheumatoid arthritis, cancer, non-hemorrhagic stroke, or respiratory disease [2,3,4,5].

Oral bacteria in dental plaque are the main cause of periodontal diseases [6]. The interaction between pathogenic microorganisms and the host immune response is accompanied by an increase in proinflammatory cytokines, matrix metalloproteinases [7], and reactive oxygen species [8].

The treatment of periodontal diseases is highly nonspecific, consisting of mechanical debridement of dental plaque and calculus from the teeth. Additionally, pharmacologic therapies based on antimicrobials, in particular antiseptics and local or systemic antibiotics are recommended [9]. Recent studies showed that the significant effectiveness of topical 14% doxycycline gel on oral anaerobic bacteria caused chronic periodontitis [10]. However, the long-term use of antibiotics increases the risk of bacterial antibiotic resistance [11]. It may be due to the use of sub-inhibitory concentrations of various antibiotics that affect the genotype and phenotype of microorganisms, increasing their resistance by formatting a biofilm [12].

Research in recent years has focused on searching for new herbal therapies in the preventive and therapeutic applications of periodontal diseases [13,14]. Herbal extracts contain various ingredients, e.g., flavonoids and other polyphenols, terpenes, and alkaloids, which are well known as an anti-inflammatory, antioxidative, and antimicrobial agents [15].

One of the plant materials with significant potential for use in treating periodontitis is *Scutellaria baicalensis* Georgi. It is also known as a Baikal skullcap or Chinese skullcap, and it is a perennial herbaceous plant from the Lamiaceae (Labiatae) family [16]. The potential of *S. baicalensis radix* in periodontal diseases is justified by its numerous properties, which lead to beneficial changes in inflammatory processes in the oral cavity. Firstly, it reduces inflammation by inhibiting the expression of proinflammatory mediators, such as IL-1β, IL-6, IL-8, and TNFα in gingival tissues, reduces alveolar bone destruction, and promotes the recovery of periodontal structures [17,18]. This action is associated with the presence of compounds: baicalin, baicalein, and wogonin. Baicalin in the rat model of periodontitis reduced alveolar bone loss and increased the surface fraction of collagen fibers by decreasing cyclooxygenase-2 and nitric oxide synthase proteins expression [19,20]. It promoted osteogenic activity in the human periodontal ligament and periodontal ligament cells by up-regulating the expression of osteoprotegerin (OPG) mRNA and proteins, down-regulating receptor activator of nuclear factor-κβ ligand (RANKL) mRNA and protein expression, and significantly reducing the ratio of RANKL/OPG expression [21,22,23]. Furthermore, baicalin inhibited the secretion of the IL-1β induced pro-matrix metalloprotein-1 (pro-MMP-1) and expression of MMP-3 in human gingival fibroblasts and periodontal ligament cells [24] and blocked MMP-8 release from polymorphonuclear leukocytes induced by IL-8 [25]. Moreover, baicalin possesses significant antibacterial properties against periodontal pathogenic bacteria [26]. Baicalein possesses anti-inflammatory activity to reduces mRNA and protein levels of proinflammatory cytokines such as IL-1β, TNF-α, MMP-1, MMP-2, and monocyte chemoattractant protein 1 in periodontal ligament cells by the mechanism of inhibiting the mitogen-activated protein kinase (MAPK) signaling pathway [27]. Subsequent studies indicate that baicalein may up-regulate the expression of the osteogenic markers in human periodontal ligament cells, including runt-related transcription factor 2, bone morphogenetic protein 2, osterix, and osteocalcin through the activation of the Wnt/β-catenin signal transduction pathway in the cells of periodontal ligaments [27,28]. In another study, wogonin inhibited lipopolysaccharide (LPS)-induced bone resorption through down-regulation of RANKL and up-regulation of OPG expression by blocking PGE_2_ production [29].

One of the ways to better use the health-promoting properties of plant raw materials is to obtain their systems with biopolymers, which extend the time of contact with places with changed tissues. Moreover, in the case of choosing the suitable biopolymer, we can also expect a synergy of action between the plant material and the carrier used. Chitosan is a carrier with valuable properties (such as anti-inflammatory, antibacterial, and bone regeneration) essential for treating oral diseases [30,31].

With regard to topical formulations applied to the oral cavity, hydrogels are regarded as a feasible delivery systems, which is due to their high biocompatibility, lack of irritating properties, and possibility of adjusting the polymer network degradation rates or drug release profiles, according to the desired therapeutic effect [32,33,34]. When selecting the composition of the formulations applied in the periodontal space, several important aspects should be taken into account, such as ease of application, entire filling of the treated area, and sufficiently long residence time [35,36]. All of the above-mentioned can be achieved by application of the thermosensitive poloxamers with a unique ability to reverse thermal gelation. This means that at lower temperatures, they are liquid, and as the temperature increases, they thicken to a semi-solid form [37,38]. Therefore, it is reasonable to use poloxamer-based vehicles, which are liquid during application, and after filling of the periodontal space, they increase the viscosity to ensure a longer residence time in the treated area. Unfortunately, due to the relatively quick dilution of poloxamer semi-solid systems with saliva, it is advisable to use additional stabilizing polymers. One of the possibilities is the addition of ion-sensitive polymers such as sodium alginate or gellan gum. Due to the presence of specific functional moieties, these polymers, in the presence of cations, including those present in saliva, can undergo cross-linking, which results in stiffening of the structure, makes it more resistant, and thus extends the residence time at the site of administration [39,40,41]. Another approach is to enhance the contact between the preparation and the tissue by introducing mucoadhesive polymers that temporarily connect with the mucosa, e.g., modified celluloses such as hydroxyethyl cellulose, methyl cellulose, hydroxypropyl cellulose, and hydroxymethyl propyl cellulose [42,43,44].

The aim of this work was to investigate the impact of chitosan on the biological behavior of *S. baicalensis radix* lyophilized extract in terms of their potential application in the treatment of periodontal diseases. For this purpose, two types of chitosan that differ in viscosity were employed in order to obtain binary mixtures of *S. baicalensis radix* extract and chitosan. Additionally, a particular effort was made toward the development of hydrogel formulations comprising thermosensitive and mucoadhesive polymers as a convenient platform for the periodontal pocket delivery of a binary mixture of *S. baicalensis radix* extract and chitosan. The study investigated both the physicochemical properties of the obtained systems and the estimated synergism of the biological activity of the components that were part of the system.

## 2. Results and Discussion

*S. baicalensis radix* is one of such herbal agents with proven efficacy in treating periodontal disease [45,46]. Various plant extracts or natural compounds are applied to reduce dental plaque formation and gingivitis [47,48]. Moreover, they are characterized by comparable or even greater efficacy than conventional medications used for the management of periodontitis, e.g., chlorhexidine [49,50,51]. Chitosan is a mucoadhesive, biodegradable, and non-toxic biopolymer, which due to its unique biological properties is widely explored as a multifunctional excipient in pharmaceutical technology [52].

### 2.1. Preparation of S. baicalensis radix Lyophilized Extract and Its Analysis

#### 2.1.1. Extract Preparation

The first work stage was to prepare a freeze-dried hydro-alcoholic (6:4, *v/v*) extract of *S. baicalensis radix*. The dried roots of *S. baicalensis* used for these studies met the pharmacopoeial requirements regarding content baicalin (9.58 ± 0.06%) [53]. The choice of appropriate solvent composition can increase the extraction efficiency of active compounds from the plant material. A previous study showed that the extraction of major flavones from *S. baicalensis radix* using 60% ethanol was the most effective [54].

#### 2.1.2. Determination of Flavonoids Content

**Figure 1:**
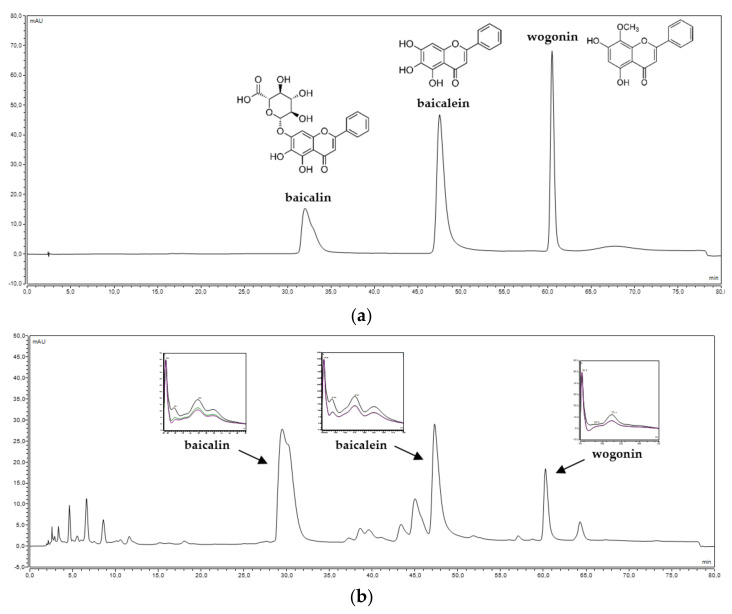
Spectral and chromatographic analysis of (**a**) baicalin, baicalein, and wogonin standards and (**b**) *S. baicalensis radix* lyophilized extract.

*S. baicalensis radix* lyophilized extract was standardized from the content of flavones by using the HPLC-DAD method. The chromatographic method developed by Wang et al. [55] was validated following the protocol issued by ICH Q2(R1) for linearity, precision, limits of detection, and quantification of analytical standards (Table S1 in Supplementary Materials) [56]. Under developed chromatographic conditions, we obtain a separation of baicalin (baicalein 7-*O*-glucuronide), baicalein, and wogonin at 30.95, 47.36, and 60.41 min, respectively. The chromatographic parameters of the separated peaks are presented in Figure 1. The content of baicalin was 178.10 ± 1.90 mg per 1 g of lyophilized extract, and the content of baicalein was 62.93 ± 1.23 mg per 1 g of lyophilized extract, while that of wogonin was 25.31 ± 0.19 mg per 1 g of lyophilized extract.

### 2.2. Preformulation Studies of S. baicalensis radix Lyophilized Extract with Chitosan and the Evaluation of Their Activity

#### 2.2.1. Binary Mixtures Preparation

*S. baicalensis radix* lyophilized extract was mixed carefully in the mortar with chitosan different in viscosity (500 and 1000 mPas) in equal amounts (*w/w*) to formulate binary mixtures (SBE/Cs 80:500 and SBE/Cs 80:1000, respectively).

#### 2.2.2. ATR-FTIR Spectroscopy

**Figure 2:**
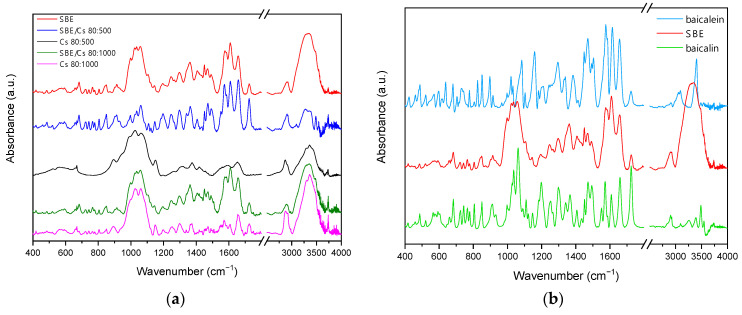
ATR-FTIR spectra of (**a**) binary mixtures and chitosans; (**b**) *S. baicalensis radix* extract, baicalin, and baicalein.

Binary mixtures of *S. baicalensis radix* extract and chitosan were characterized by Fourier transform infrared spectroscopy (FTIR) using the attenuated total reflectance technique (ATR). The results of FTIR spectral analysis are demonstrated in Figure 2. The observed characteristic bands for flavones are consistent with the literature [57,58,59]. Characteristic bands of *S. baicalensis radix* lyophilized extract at 3333 cm^−1^, 1726 cm^−1^, and 1657 cm^−1^ were attributed to the stretching vibration of the O-H, -COOH, and C=O groups in flavones, respectively. The signals at 1609 cm^−1^, 1584 cm^−1^, and 1576 cm^−1^ were induced by the C=C vibration stretching of the aromatic rings in the flavones. The signals in the range 1200–900 cm^−1^ were attributed to the various stretching vibrations of C–O bonds of saccharides. The peak at 1059 cm^−1^ was characteristic for the stretching vibration of glycosidic bonds (C-O-C) in flavonoids glycosides. The FTIR spectra of chitosan showed characteristic bands at 3362 cm^−1^ and 3298 cm^−1^ due to the stretching vibrations of O-H and N-H groups. The absorption peaks at 2920 cm^−1^ and 2872 cm^−1^ are due to symmetric and asymmetric C-H stretching, and the absorption peaks at 1657 cm^−1^ and 1570 cm^−1^ can be attributed to N-H bending vibration. The stretching vibration of CH_3_ and CH_2_ groups was attributed in the range of 2800–3000 cm^−1^ and 1300–1500 cm^−1^. In the range 900–1200 cm^−1^ were shown the bands of peaks for the following bonds: 1150 cm^−1^ (asymmetric stretching of C-O-C bridge), 1059 cm^−1^ (C-O stretch, secondary hydroxyl group), and 1026 cm^−1^ (C-O stretch, primary hydroxyl group). The peak at 1150 cm^−1^ was characteristic of glycoside linkages [60,61,62,63]. These results illustrated that no change occurred in the chemical structure of the *S. baicalensis radix* extract after being mixed with chitosan.

#### 2.2.3. Dissolution Studies

**Figure 3:**
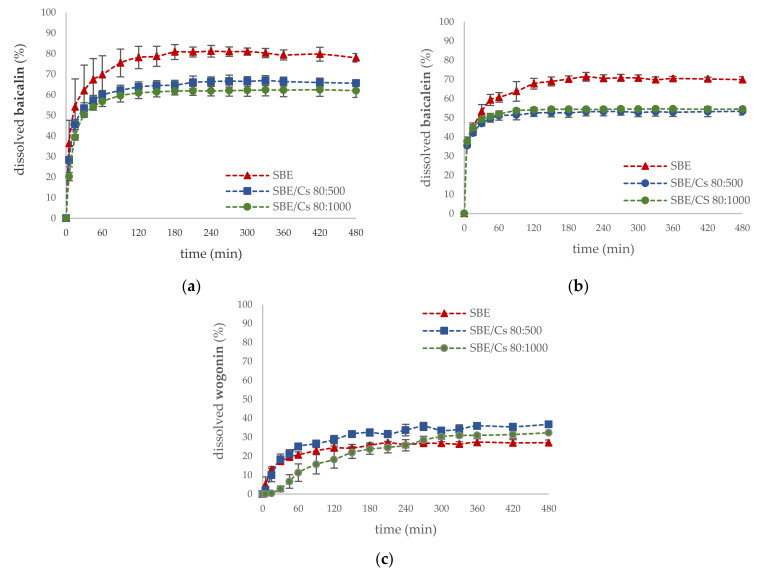
Dissolution profiles of (**a**) baicalin; (**b**) baicalein; (**c**) wogonin of *S. baicalensis radix* extract and binary mixtures in pH 6.6.

In order to provide information on the rate and extent of the release of active compounds from binary mixtures with *S. baicalensis radix* extract, dissolution studies were conducted. Results from the dissolution studies showed that the formulations SBE/Cs 80:500 and SBE/Cs 80:1000 were prolonged the release rates of baicalin (65.65 ± 1.58% and 62.03 ± 3.28%, respectively) and baicalein (53.32 ± 1.94% and 54.48 ± 0.43%, respectively) compared with *S. baicalensis radix* extract at the end of the 480 min dissolution studies, as shown in Figure 3a,b. Surprisingly, after mixing the lyophilized extract with chitosans, a slight increase in the dissolution rate of wogonin was observed (Figure 3c). The previously published studies indicated that chitosan is a proper carrier for poorly water-soluble drugs [61,64].

#### 2.2.4. Permeability Studies

In vitro permeability of active compounds from binary mixtures was carried out using a parallel artificial membrane permeability assay (PAMPA-GIT) in pH 6.6, simulating the pH of the saliva. The systems were equilibrated for 4 h for baicalin and 1 h for baicalein and wogonin at 37 °C, based on literature values of permeability [65]. As shown in Table 1, among the studied standards, only baicalein and wogonin (P*_app_* (91.56 ± 2.72) × 10^−6^ cm s^−1^ and (57.23 ± 5.42) × 10^−6^ cm s^−1^, respectively) were characterized by high passive diffusion across the plasma lipid membrane. Moreover, the higher values of permeability of these compounds from *S. baicalensis radix* extract and binary mixtures were observed in comparison to standards alone. This beneficial effect may be due to the presence of other compounds in the extract such as fatty acid [66], which are considered to be mucosal permeation enhancers [67]. On the other hand, the low permeability of baicalin (P*_app_* (0.02 ± 0.01) × 10^−6^ cm s^−1^) resulted from its relatively high hydrophilicity and large molecular weight [68] and absorption in the form of an aglycone after hydrolysis by intestinal bacteria [69].

#### 2.2.5. Biological Activity

The biological activities of binary mixtures of lyophilized *S. baicalensis radix* extract and chitosan were assessed from methodologies in vitro, evaluating the possibilities of inhibition degradation of hyaluronic acid and the potential of antioxidant and antimicrobial activity.

**Figure 4:**
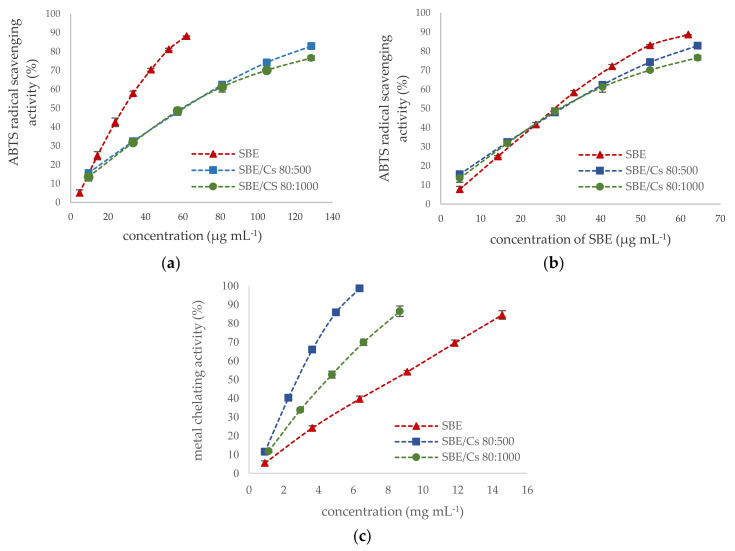
Concentration-dependent antioxidant activity of *S. baicalensis* extract and binary mixtures. (**a**) ABTS assay calculated per µg of SBE and binary mixtures (mean ± S.D., *n* = 6); (**b**) ABTS assay calculated per µg of SBE in binary mixtures; (**c**) metal-chelating activity (mean ± S.D., *n* = 6).

Several herbal extracts or natural compounds play an important role in treating various disease symptoms of periodontitis diseases due to their antioxidant properties [70,71]. Free radicals and reactive oxygen species play an essential role in an increased inflammatory response in the pathogenesis of periodontitis, which leads to the destruction of periodontal tissues and alveolar bone [8,72]. Antioxidant activity of *S. baicalensis radix* extract and binary mixtures were investigated by using two methods of different mechanisms, such as ABTS^•+^ radical cation assay and ferrous ion-chelating assay. Due to the multifunctional properties of natural antioxidants, these methods are widely employed to evaluate the antioxidant activity of plant extract [73,74]. ABTS assay utilized the SET (*single electron transfer)* mechanism. In contrast, metal chelating prevents the generation of reactive oxygen species in the Fenton and Haber–Weiss reaction by forming complex structures of antioxidants with transition metals (mainly iron and copper) [75]. Moreover, previous reports documented the positive correlation between the levels of transition metal ions in periodontal pockets and periodontitis severity [8]. As shown in Figure 4a, the *S. baicalensis radix* lyophilized extract showed twice higher the scavenging activity against the radical cations ABTS^•+^ (IC_50_ = 28.49 µg mL^−1^) than the binary mixtures (IC_50_ = 60.59 µg mL^−1^ and IC_50_ = 59.88 µg mL^−1^ for chitosan 80:500 and 80:1000, respectively). The ABTS radical scavenging activity of the binary mixtures resulted only from the presence of *S. baicalensis radix* lyophilized extract (Figure 4b). Similar results were obtained for encapsulated chlorogenic acid [76]. The crucial role in the scavenging activity of the *S. baicalensis radix* extract is the presence of flavonoids with numerous phenolic hydroxyl groups [77], whereas chitosan has an insignificant radical scavenging activity due to insufficient H-atom donors [78]. On the other hand, the metal-chelating capacity of binary mixtures was higher than that of *S. baicalensis* extract (IC_50_ = 8.32 µg mL^−1^), and the chitosan viscosity significantly influenced forming complexes of iron (III) with chelators with IC_50_ values 2.79 µg mL^−1^ or 4.52 µg mL^−1^ for 500 and 1000 chitosan viscosity, respectively (Figure 4c). The higher antioxidant activity of lower viscosity chitosans was also demonstrated in preventing lipid oxidation in the herring flesh model system [79]. The hydroxyl groups (OH) and amino groups (NH_2_) in the chitosan molecule are the crucial factor of its chelating properties [80]. Additionally, the *S. baicalensis radix* is known to have a strong complexing ability through interactions 6,7-dihydroxy structure of flavonoids (especially baicalein) with transition metals [81,82].

Hyaluronic acid is a naturally occurring glycosaminoglycan of the extra-cellular matrix of the periodontal ligament [83]. Hyaluronic acid plays a major role in periodontal tissue differentiation and proliferation binding to membrane receptor CD44 as well as reduces local inflammatory processes and alveolar bone resorption by inducing the up-regulation of inflammatory genes in tissues affected by periodontitis [84,85,86]. Moreover, hyaluronan can reduce the colonization and proliferation of pathogenic bacteria in the gingival crevice and adjacent periodontal tissues [87]. During periodontal diseases, hyaluronic acid can be extensively depolymerized to fragments with lower molecular weight by the activity of hyaluronidases, β-glucuronidases, hexosaminidases, and reactive oxygen species [88,89]. Low molecular weight hyaluronan possessing proinflammatory activity has been reported to be present in the gingival tissue of patients at the initial phase of periodontal disease [83,90]. Plant extracts and natural compounds are known to be hyaluronidase inhibitors [91,92,93]. As shown in Figure S1 (Supplementary Materials), binary mixtures had stronger activity (IC_50_ 0.12 mg mL^−1^ and 0.18 mg mL^−1^ for chitosan 80:500 and 80:1000 respectively) than *S. baicalensis radix* extract alone (IC_50_ = 2.19 mg mL^−1^). A significant increase in the inhibition of hyaluronic acid degradation may point toward a synergistic action of active compounds from *S. baicalensis* extract, particularly baicalein [94,95,96] and chitosan molecules [97]. A similar result was obtained by Mao et al. for chitosan oligosaccharide modified by grafting linalool [98].

The potential against bacterial and fungal species inhabiting the oral cavity of humans for them was also evaluated. Plant species play a vital role in inhibiting pathogen growth and invading gingival tissue [15,99]. *S. baicalensis radix* [100,101] and chitosan [30,31,102,103] are well-known antimicrobial and antifungal agents. The antimicrobial activity of lyophilized extract and binary mixtures was evaluated according to their minimum inhibitory concentrations (MICs) against various pathogens: four species of Gram-positive bacteria (*Staphylococcus aureus*, *S. epidermidis*, *Streptococcus mutans*, *Actinomyces naeslundii*), three Gram-negative bacteria (*Escherichia coli*, *Proteus mirabilis*, *Prevotella intermedia*), and two species of yeast-like fungi (*Candida albicans, C. tropicalis*). Antimicrobial activity against Gram-positive lactic acid bacterium *Lactobacillus acidophilus* was also carried out. Used in this study as solvent, a 20% water solution of dimethyl sulfoxide DMSO did not present antibacterial and antifungal activity. The results showed a significant increase in the sensitivity of pathogens to the *S. baicalensis radix* extract with chitosan 80:500 compared to the lyophilized extract and flavones (Table 2). Most sensitive to its effects were Gram-positive bacteria and yeast-like fungi, which is inconsistent with previous reports [102,104]. A binary mixture with chitosan 80:500 exhibited low antimicrobial efficacy or was inactive against *P. mirabilis*, *P. intermedia*, and *A. naeslundii* with MICs in the range of 1250 and >2500 μg mL^−1^. Binary mixture with chitosan 80:1000 was found inactive against all bacteria and fungi tested.

Preformulation studies indicated the stronger biological activity of innovative binary systems compared to pure *S. baicalensis radix* lyophilized extract toward anti-hyaluronidase, metal chelating, and antimicrobial activities. Moreover, the presence of chitosan allowed for the prolonged release of flavones baicalin, baicalein, and wogonin, without affecting the permeation of active compounds through membranes simulating the gastrointestinal tract from the binary mixtures.

### 2.3. Formulation Studies Involving S. baicalensis radix Lyophilized Extract with Chitosan and the Evaluation of Its Quality

#### 2.3.1. Preparation of the Formulations and Rheological Analysis

Rheological analysis plays a key role in developing the composition of semi-solid formulations, including those applied in the oral cavity [105]. Properly designed rheological properties translate into the conditions of the production process, the method of application, and may also correlate with the release of the active ingredient and the therapeutic efficacy [106]. In this study, the properties of thermo-sensitive hydrogels gelling in situ in the periodontal space based on poloxamer 407 were developed and assessed. The assumption was to obtain a formulation with a liquid consistency at room temperature, which would fill it up and then thicken when introduced to the target site. Sodium alginate, an ion-sensitive mucoadhesive polymer capable of reacting with saliva ions, was used as a component that stiffens the polymer network. In addition, it was used to assure proper rheological properties. For the preparation of the formulations, a binary mixture with *S. baicalensis radix* extract and chitosan 80:500 (SBE/Cs 80:500) was used due to its higher antioxidant, anti-hyaluronidase, and antimicrobial activities. Hydrogels loaded with 2 or 4% of binary mixture and a corresponding placebo formulation were analyzed for steady shear by plotting flow curves. The thermal solidification process was also assessed to determine the gelation temperature (Tsol–gel). In order to more precisely define the microstructure of the tested formulations, an advanced oscillatory analysis was performed, including stress (amplitude) sweeping stress and frequency.

*Flow curves.* In the steady-shear experiments, the samples were subjected to the increasing shear rate, and the shear stress was monitored. The obtained data were plotted as the flow curves (Figure S2 in Supplementary Materials). As can be observed, the shape of the curves indicates the shear-thinning properties preceded by rapid breakage of the polymer structure typical for hydrogels containing poloxamer 407, not showing the ductility that characterizes most natural polymers. In the case of formulations containing MC, the curves showed the best fitting to the Ostwald de Waele (power law) model:

$$\tau =K\cdot {\dot{\gamma }}^{n}$$

where *n* is a power law index and represents the fluidity [107]. In general, it can be assumed that the more the value of *n* differs from unity, the more the properties of the tested system differ from the Newtonian behavior (*n* = 1). The *n* parameter illustrates the degree of the structure of the gel changes with the increase in the shear rate. The *K* parameter K is referred to as the consistency coefficient. Its value corresponds to the shear stress at a shear rate of 1.0 s^−1^. The power law can be applied to materials with a strong internal structure. As presented in Table 3 for the mentioned MC gels, the *n* values are very low, which indicate their typical shear-thinning nature. As can be seen, addition of the binary mixture with. *S. baicalensis radix* extract had no effect on *n*, regardless of its percentage content. On the other hand, in the case of the *K* value, the addition of 2% did not affect its value, while at 4%, a clear decrease can be noticed. In the case of a placebo sample containing HPC, the curves showed best fitting to the Herschel–Bulkley model, which is used to describe materials that follow the power law flow behavior but reveal the presence of yield stress (τ_0_) [108]. Despite the high value of τ_0_, the above-mentioned samples showed lower *K* values than the MC samples, while the *n* values were switched closer to 1.0, which suggests a tendency to a more plastic reaction of the tested system. Surprisingly, the addition of the binary mixture to an HPC containing vehicle resulted in a lower reproducibility of the results and irregular shape of the flow curves, which are probably related to the varied shape of the binary mixture particles. Therefore, it was impossible to obtain reliable data and to fit the curves to the appropriate model. It can be stated that with the presence of HPC in formulation, the consistency index decreased to a large extent. Moreover, in the case of MC containing formulations, an addition of 2% of the binary mixture did not have any visible effect on the consistency, but increasing its amount to 4% contributed to decreasing this parameter.

*Temperature sweeping.* The evaluation of the thermosensitivity of the systems was assessed in steady shear conditions with a constant shear rate (10 1 s^−1^) and increasing temperature. Two parameters were determined from the shape of the curves, such as Tsol–gel corresponding to the average viscosity value during the entire measurement and the maximum viscosity obtained by the formulations during the test. As can be seen in Figure S3 (Supplementary Materials) and Table 3, the gelation temperature of all formulations was within the range of 25–27 °C. In comparison to MC samples, HPC contributed to a slight decrease of about 1 °C. Among MC gels (formulations F1-2 and F1-4), it could be seen that the presence of the binary mixture did not have any influence on Tsol–gel. Additionally, it can be stated that placebo sample P1 showed a more rapid gelation process, whereas after binary mixture addition, the gelation process was more extended.

**Figure 5:**
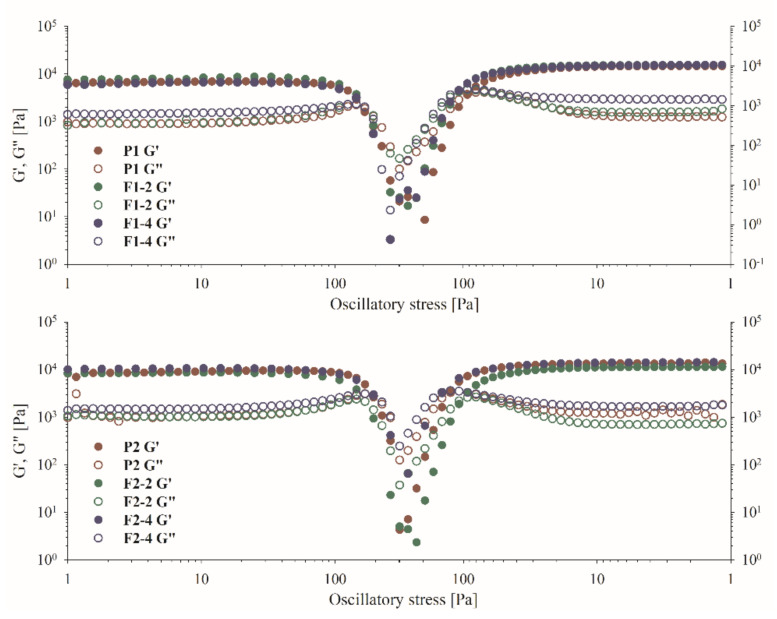
The oscillatory stress sweeping of the gel samples.

*Dynamic oscillatory experiments.* Oscillatory measurements were performed in two modes, the first in stress (amplitude) sweeping (SS) and the second in frequency sweeping (SS) [109]. The SS analysis was carried out in two stages, under increasing and decreasing stress, due to depicting the ability of the samples to rebuild their structure after destruction. The samples were subjected to the increasing stress, which changed sinusoidally. Changes in the values of the storage (G′) and loss (G″) were monitored. The value of angular frequency was constant and equal to 1 Hz (6.2832 rad s^−1^). On the basis of the obtained results, the LVR (Linear Viscoelastic Regime) of the tested samples was first defined. LVR depicts the stress range during which the internal structure remains intact and both moduli are independent on the stress. As it was shown in Figure 5, all of the formulations revealed typical elastic behavior as G′ prevailed G″. The LVR also depicts the mechanical stability, which is the result of a well-packed and linked polymer structure. Broad LVR also characterizes well-dispersed materials. As presented on the plots, the values of both modules began to approach to each other under the influence of increasing stress, mostly as the result of polymer chains extension. The cross-over point of G′ and G″ depicts the moment of elasticity loss, beyond which the samples behave more as liquids (non-linear region). As it was mentioned, the samples were subjected to increasing and decreasing stress, and two cross-over points were determined respectively, one for breakage of the structure and the second depicting its reconstruction. According to the calculated rheological data presented in Table 3, a P1 sample revealed shorter LVR than P2, which could suggest that HPC contributed to stiffening of the structure. However, such a relationship was observed only for the placebo samples, whereas after addition of the binary mixture, the polymer-related effects were not visible. It was noticed that in the presence of the *S. baicalensis radix* extract with chitosan 80:500, the structure of the HPC-containing gels liquefied, whereas this effect was not observed for MC gels. An interesting observation concerned the structure recovery of the gels. In all cases, the breakdown stress values were higher than those analogous at the recovery stage. It shows that the intact gels had the ability to more strongly counteract the deforming force due to the stabilization of the polymer network and the interactions between the components. In case of the rebuilding process, the return of the mechanical strength was faster, and the obtained values of both modules were higher than the initial values.

After measurement of the LVR values, the samples were subjected to increasing frequency under constant stress. The frequency sweeping provides essential information on the structure and nature of a given material and how the microprocesses, colloidal forces, and interactions translate into external mechanical properties. The rate of G′ dependency on the frequency shows how the delivered energy is stored or dissipated throughout the material. The fluidity of a given material increases with the frequency dependency. Figure S4 (Supplementary Materials) presents the values of G′, G″, and complex viscosity as a function of frequency. The value of G′ prevailed G″ in the range of applied frequencies. This indicates that the gels are solid-like and can be described as well-structured. Moreover, G′ showed only a narrow dependency on the frequency, which confirmed the previous observations. However, from the dependence of complex viscosity on the frequency, it can be concluded that all samples showed the evidence of viscosity loss. Changes in complex viscosity depict how viscous and elastic properties influence the flow behavior of given material [110]. It is also defined as the total resistance to flow as a function of angular frequency [111]. As can be seen in Figure S4, the complex viscosity of all samples showed a dependence on the oscillation frequency, and its values decreased with increasing frequency. It can be concluded that despite the existence of a three-dimensional polymer network, typical for structured gels, the tested formulations are able to resist slight deformation forces but are easily destroyed when the forces increase and their structure becomes more liquid-like.

In general, it can be considered that the developed gels meet the requirements regarding the mechanical properties of preparations for use in the oral cavity and will constitute suitable vehicles for the tested active ingredients.

#### 2.3.2. In Vitro Dissolution Studies

**Figure 6:**
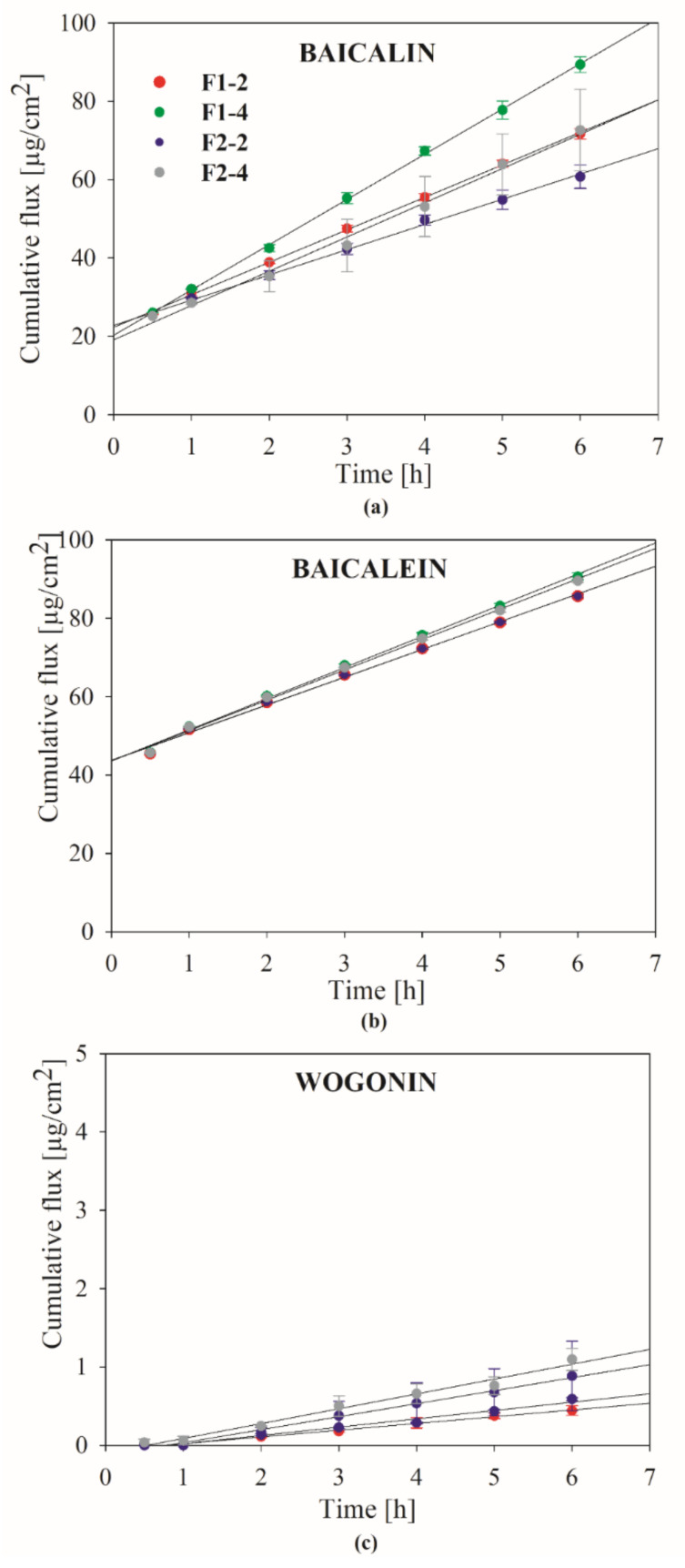
Dissolution profiles of (**a**) baicalin; (**b**) baicalein; (**c**) wogonin from hydrogels with binary mixture with *S. baicalensis radix* lyophilized extract and chitosan using acceptor solution (pH 6.6) containing potassium chloride, sodium chloride, di-potassium hydrogen orthophosphate, magnesium chloride, calcium chloride, and xylitol.

Studies with the use of Franz diffusion cells were carried out to assess in vitro release profile of three active ingredients, baicalin, baicalein, and wogonin. The obtained results are presented in Figure 6 and Table S2 (Supplementary Materials) as the dependence of cumulative flux (*Jss*) on time. When using the infinite dose conditions, the permeation of the active compounds in a steady-state period follows Fick’s first law. It has to be taken into account that in the case of artificial membranes, the process undergoes a slightly different pattern, which is related to the fact that the membrane does not constitute a tight border; therefore, it can be used to simulate the conditions present after application of the formulation to the periodontal pocket, as it does not have the direct impact on the active compounds release. Within the steady-state period, the flux (*Jss* = d*Q*/d*t*, µg cm^−2^ h^−1^) is constant and can be calculated as the slope of the linear regression of the released amount as a function of time. This value can be further used to calculate the release coefficient *Kr* = *Jss/C_0_* [112,113]. As can be seen from the course of the release profiles, this process, in the measured range, followed the zero-order release kinetics. However, it must be emphasized that due to the fact that the formulations were poured to the donor compartment of the Franz cell in a liquid state, a very fast onset of the release process could have occurred. The sample dosing started after 30 min of the experiment; therefore, some amounts of the actives could be released immediately after application and before thickening of the vehicle. It also has to be kept in mind that after mixing of the binary mixture with the vehicles, some amounts of the active compounds that were not bound to the chitosan particles rapidly dissolved, and this fraction could immediately diffuse across the membrane. Nevertheless, in the case of baicalin and baicalein, the profiles suggest that throughout the experiment, the release was not diffusion dependent. Probably, it was also the effect of gradual water uptake dilution of the gel formulation in acceptor fluid. Wogonin displayed the slowest release rate, and the lowest cumulative amounts of this compound at the end of the studies were observed in acceptor media. Moreover, for this compound, a 1 h release lag time period was noticed. According to baicalin and baicalein, it was clearly shown that the *Jss* and *Q_6h_* values increased with the amount of the binary mixture in the formulation. In both cases, the *Kr* values had the highest values for the binary mixture concentration of 2%. Baicalin showed a slower release rate from the formulation F2 (with HPC), but the effect was not noticeable for baicalein. It can be also stated that baicalein was released in larger amounts than baicalin, as the values of *Q_6h_* were predominantly higher. The parameters calculated for wogonin show that the release was significantly slower and time extended than the other two substances. It should be taken into account that one of the components present in the media was PEG400, which may act as a co-solvent for lipophilic substances. The applied components of the vehicle, due to the presence of active ingredients, both in free form and bound to the chitosan carrier, have the potential to provide a two-stage release, with the first initial dose and the subsequent maintenance dose.

It should be emphasized that the results obtained on the Franz cells do not have to directly correlate with the PAMPA penetration test. In the first case, the applied synthetic diffusion membranes act only as a mechanical border to the formulation and its components and do not interact directly with active compounds molecules; hence, the process that takes place is called release. In the case of the PAMPA test, the composition of the membrane determines the permeation of active compounds depending on their physicochemical properties, and thus, the process can take a different course.

It can be stated that the slower the release of the active compounds, the better, especially bearing in mind that in the oral cavity, the gels will have direct contact with saliva and will be exposed to oral movements, which to some extent may accelerate the degradation of the polymer network and can liquefy their structures.

#### 2.3.3. Mucoadhesive Properties

**Figure 7:**
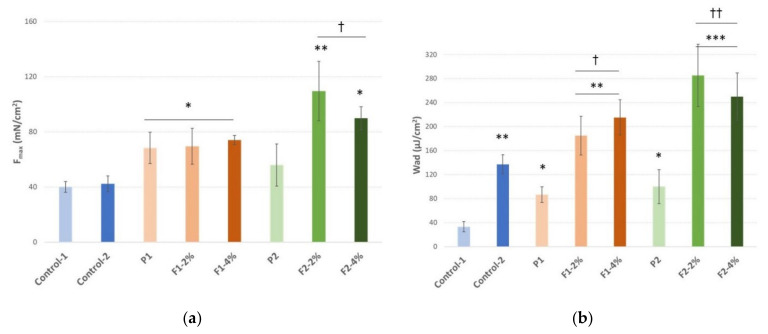
(**a**) Maximum detachment force (F_max_) expressed in millinewtons (mN); (**b**) work of mucoadhesion (W_ad_) expressed in microjoules per tissue area unit (µJ/cm^2^) of hydrogels with a binary mixture of lyophilized extract of *S. baicalensis radix* and chitosan 80:500 (in weight ratio 1:1) (F1–F2), placebo formulations (P1–P2), and controls (Control-1 cellulose paper; Control-2 commercial oromucosal gel) in contact with excised porcine cheek (mean ± S.D., *n* = 5); * represents substantial differences with *p* ≤ 0.05, ** with *p* ≤ 0.01 and *** with *p* ≤ 0.001 in comparison to Control-1; † and †† symbolize significant differences with *p* ≤ 0.05 and *p* ≤ 0.01 in comparison to placebo formulations.

Ex vivo tensometric analysis measured the maximum detachment force (mimicking the mechanical stress caused by e.g., sharp tongue movements interrupting contact between the formulation and buccal tissue) and the work of mucoadhesion (imitating the overall ability to retain on the buccal epithelium after application) required to separate the tested formulations; the placebo from the excised porcine cheek displayed anatomical and structural resemblance to human buccal epithelium [114]. Both bases P1 and P2 used for hydrogel formation comprise hydrophilic polymers (Table 4) commonly regarded as mucoadhesive materials [115]. As expected then, all examined formulations displayed substantial mucoadhesive behavior when compared to negative control but responded differently upon contact with excised porcine cheek (Figure 7).

The presence of a binary mixture of *S. baicalensis radix* extract and chitosan improved the mucoadhesive capacity of formulations. In fact, hydrogels F1-F2 exhibited a greater ability to interact with porcine cheek as compared to the values obtained with the reference commercial oromucosal gel. Notably, the strength of the mucoadhesive bond was not altered by increasing the concentration of binary mixture from 2% to 4% in formulations F1 and F2.

The polymer composition and the type of cellulose derivative used in the designed formulations influenced their mucoadhesiveness upon the addition of a mixture of *S. baicalensis radix* extract and chitosan. Basically, formulations F2 containing hydroxypropyl cellulose demonstrated greater Fmax and Wad values than those observed for formulations prepared of methylcellulose (F1). In turn, no real differences in mucoadhesiveness were noticed when compared placebo formulations P1 and P2.

## 3. Materials and Methods

### 3.1. Preparation of S. baicalensis radix Lyophilized Extract and Its Analysis

#### 3.1.1. Extract Preparation

The roots of *Scutellaria baicalensis* Georgi were purchased from the NANGA, Zlotow, Poland (Lot No. 243112019). Total baicalin content in the plant material was determined by using UPLC-DAD method. According to Ph. Eur. 9th Edition, the dried root of *S. baicalensis* should contain baicalin not less than 9.0% [53].

Five hundred grams of dried *S. baicalensis* roots were extracted three times with ethanol–water (6:4), each time for 90 min at 95 °C in a water bath. The obtained extract was concentrated under the vacuum (BÜCHI Rotavapor R-210, Büchi Labortechnik GmbH, Essen, Germany) at a temperature below 40 °C to a syrupy consistency, frozen, and then lyophilized. The freeze drying was conducted at reduced pressure (2–9 hPa) at a condensation temperature of −55 °C for 48 h (Heto PowerDry PL3000, Thermo Fisher Scientific, Waltham, MA, USA). Then, 192.67 g of lyophilized extract from *S. baicalensis* roots (SBE) were obtained.

#### 3.1.2. Determination of Flavonoids Content

The determination of flavones (baicalin, baicalein, wogonin) in the lyophilized extract of *S. baicalensis radix* was performed according to the High-Performance Liquid Chromatography with diode array detector (HPLC-DAD) method developed by Wang et al. [55]. The HPLC system comprised a high-performance liquid chromatography (DionexThermoline Fisher Scientific, Waltham, MA, USA) equipped with a high-pressure pump (UltiMate3000), an autosampler (UltiMate 3000), and a DAD detector (UltiMate 3000). Analyses were performed on a Luna C18(2) column (5 μm, 4.60 mm × 250 mm, Phenomenex). The linear gradient was as follows: 42–43% B over 0.0–18.0 min, 43–46% B over 18.0–30.0 min, 46–50% B over 30.0–45.0 min, 50–58% B over 45.0–55.0 min, 58–61% B over 55.0–65.0 min, 61% B over 65.0–75.0 min, and 42% B over 75.0–80.0 min with a flow rate of 1.0 mL min^−1^ at the column temperature 30 °C. The chromatographic profile was recorded at 280 nm. The injection volume of the sample was 5.0 μL. As a mobile phase, 0.1% acetic acid (eluent A) and methanol (eluent B) were used. Quantification of flavonoids in *S. baicalensis radix* lyophilized extract and binary mixtures was performed using Chromeleon software version 7.0 comparing the peak area with standard reference curves (10–250 μg mL^−1^).

The HPLC-DAD method was validated according to the International Conference on Harmonization Guideline Q2 (ICH) for linearity, precision, the limit of detection, and the limit of quantification (LOD and LOQ, respectively) [56].

### 3.2. Chemicals and Reagents

Baicalin, baicalein, and wogonin as phyproof^®^ reference substances, as well as sodium alginate—AlgNa, methyl cellulose—MC, poly(ethylene glycol)—PEG400 (M.W. 400), poloxamer 407—P407 (Kolliphor^®^ P407), and 2-hydroxypropyl cellulose—HPC were purchased from Sigma-Aldrich Co. (St Louis, MO, USA). Chitosan (Cs) 80:500 (degree of deacetylation: 77.6–82.5%; viscosity: 351–750 mPas in 1% acetic acid at ambient temperature) and 80:1000 (degree of deacetylation: 77.6–82.5%; viscosity: 751–1250 mPas in 1% acetic acid) were supplied from Heppe Medical Chitosan GmbH (Halle, Germany). High-quality pure water and ultra-high-quality pure water were prepared by using a Direct-Q 3 UV Merck Millipore purification system. Solvents used for the determination of total flavonoid content and HPLC method were purchased from Avantor Performance Materials Poland S.A. (Gliwice, Poland). All other chemicals were from Sigma–Aldrich Chemical Co.

### 3.3. Preformulation Studies of S. baicalensis radix Lyophilized Extract with Chitosan and the Evaluation of Their Activity

#### 3.3.1. Binary Mixtures Preparation

The standardized *S. baicalensis radix* lyophilized extract was mixed in an agate mortar for 45 min with two types of chitosan with a degree of deacetylation of 80% and different viscosity (500 mPas and 1000 mPas) in a weight ratio of 1:1 (*w/w*) to obtain binary mixtures (SBE/Cs 80:500 and SBE/Cs 80:1000, respectively) as uniform powders. The obtain binary mixtures were stored at room temperature.

#### 3.3.2. ATR-FTIR Spectroscopy

The molecular characteristics of binary mixtures were confirmed using Attenuated Total Reflectance spectroscopy (ATR-FTIR) using a Bruker Equinox 55 spectrometer (Bruker Optics, Ettlingen, Germany). The spectra were recorded with 400 scans in a range between 4000 and 400 cm^−1^ at a resolution of 4 cm^−1^ by using x software.

#### 3.3.3. Dissolution Studies

The dissolution profiles of active compounds from binary mixtures were determined in 150 mL of phosphate buffer solution at pH 6.6 prepared according to Ph. Eur. at 50 rpm in a standard paddle Agilent 708-DS Dissolution at 37 ± 0.5 °C for 24 h [116]. Sink conditions were maintained throughout the studies. In all experiments, 2.0 mL dissolution samples were collected at appropriate time intervals and replaced by equal volumes of temperature-equilibrated media and filtered through a 0.45 μm membrane filter. For the quantification of flavones, the UHPLC-DAD method was used. The similarity of dissolution percentage of active compounds from binary mixtures was established based on *f*_1_ and *f*_2_ parameters and was defined by the following equation:

(1) $$f_{1}=\frac{\sum_{j=1}^{n}\left|R_{j}-T_{j}\right|}{\sum_{j=1}^{n}R_{j}}\times 100$$

(2) $$f_{2}=50\times \log\left({\left(1+\left(\frac{1}{n}\right)\overset{n}{\underset{j=1}{\sum }}{\left|R_{j}-T_{j}\right|}^{2}\right)}^{-\frac{1}{2}}\times 100\right)$$

in which *n* is the number of withdrawal points, *R*_j_ is the percentage dissolved of reference at time point *t*, and *T*_j_ is the percentage dissolved by test at time point *t*. The *f*_1_ value close to 0 and *f*_2_ value close to 100 indicate profile similarity [117].

#### 3.3.4. Permeability Studies

The permeability of active compounds from binary mixtures was investigated using the PAMPA GIT model according to the method described by Paczkowska et al. [118]. The standards, *S. baicalensis radix* extract, and binary mixtures were dissolved in the donor solution adjusted to pH 6.6. All plates were incubated for 1 h and 4 h at 37 °C. The concentration of permeated flavones was determined using the HPLC-DAD method. The apparent permeability coefficients (P*_app_*) were calculated from the following equation:

(3) $$P_{app}=\frac{-ln\left(1-\frac{C_{A}}{C_{equilibrium}}\right)}{S\times \left(\frac{1}{V_{D}}+\frac{1}{V_{A}}\right)\times t}$$

where *V*_D_—donor well volume (0.2 mL), *V*_A_—acceptor well volume (0.2 mL), *C*_equilibrium_—equilibrium concentration $C_{equilibrium}=\frac{C_{D}\times V_{D}+C_{A}\times V_{A}}{V_{D}+V_{A}}$, *C*_D_—the compound concentration in the donor well, *C*_A_—the compound concentration in the acceptor well, *S*—membrane area, *t*—incubation time (in seconds).

To verify that P*_app_* determined for permeability was statistically different, an ANOVA test was used. Compounds with the value of P*_app_* < 0.1 × 10^−6^ cm s^−1^ are classified as low-permeable, compounds found as medium permeable have a value of 0.1 × 10^−6^ cm s^−1^ ≤ P*_app_* < 1.0 × 10^−6^ cm s^−1^, and compounds with a P*_app_* ≥ 1 × 10^−6^ cm s^−1^ are defined as high-permeable compounds [119]. Each experiment was performed three times.

#### 3.3.5. Biological Activity

Systems extract of *S. baicalensis radix* with chitosan were tested to evaluate their biological activity in periodontal diseases. The antioxidant potential (ABTS assay and ferrous ion chelating activity), anti-inflammatory effect (anti-hyaluronidase activity), and microbiological activity of binary mixtures compared with the *S. baicalensis radix* extract alone were estimated.

The solutions to antioxidant and anti-hyaluronidase activity studies of *S. baicalensis radix* lyophilized extract and binary mixtures were prepared by shaking (400 rpm min^−1^) with buffer solutions at pH 6.6 on a shaker (Thermo Scientific MaxQ 4450, Waltham, MA, USA) for 30 min. at 37 °C and then centrifuging at 4100 rpm min^−1^ for 20 min (Nüve NF 800, Ankara, Turkey) to produce a clear supernatant. The IC_50_ values were calculated with OriginPro 9 software. All experiments were performed six times.

##### ABTS Assay

The ABTS radical scavenging activity was conducted by the modified method of Re et al. with a slight modification [120]. The samples were diluted with the phosphate buffer to concentrations 0.1–1.3 mg mL^−1^ for *S. baicalensis radix* lyophilized extract and 0.2–2.7 mg ml ^−1^ for binary mixtures. After the addition of 200 μL of ABTS^•+^ solution to 10 μL of the sample in a well of a 96-well microplate, the absorbance was recorded at 734 nm using a spectrophotometer Thermo Scientific Multiskan GO (Thermo Fisher Scientific, Waltham, MA, USA). The control blank contained water instead of the studied solution. The percentage inhibition of the ABTS^•+^ by the test samples was calculated according to the following formula:

ABTS scavenging activity (%) = [Acontrol − Asample/Acontrol) × 100

where Acontrol is the absorbance of the control and Asample is the absorbance of the sample. The IC_50_ values, i.e., the amount of antioxidant necessary to obtain half of the initial ABTS^•+^ concentration, were used to compare the quality of the antioxidant potency of the studied extract. The lower absorbance of the reaction mixture indicated a higher free radical scavenging activity.

##### Ferrous Ion-Chelating Activity

The chelating ability was determined following the method of Dinis et al. with some modifications [121]. Briefly, 10 μL of 1 mM FeCl_2_ were added to 0.2 mL of concentrations (1.0–19.0 mg/mL in phosphate buffer pH 6.6) of the samples. The reaction was initiated by the addition of 10 μL of 2.5 mM ferrozine solution. After incubating for 30 min at room temperature, the absorbance of the mixture was measured spectrophotometrically at 562 nm. The results were expressed as a percentage of the inhibition of ferrozine–Fe^2+^ complex formation, using the following equation:

Metal chelating activity (%) = (Acontrol − Asample)/Acontrol × 100

where Acontrol is the absorbance of the control reaction (without extract or binary mixtures), and Asample is an absorbance in the presence of extract or binary mixtures. All experiments were performed six times. The IC_50_ values (50% inhibition) were calculated from the plot of chelating percentage against concentration and used for comparing the quality of the studied samples.

##### Anti-Hyaluronidase Activity

The inhibition of hyaluronidase was performed by a turbidimetric method described by Studzińska-Sroka et al. [122]. The final concentrations for binary mixtures and *S. baicalensis radix* lyophilized extract were 0.04–0.25 mg mL^−1^ and 1.6–2.4 mg mL^−1^, respectively.

##### Antimicrobial Activity

All tested substances were dissolved in 20% water solution of dimethyl sulfoxide DMSO. The minimum inhibitory concentrations (MICs) were evaluated for five Gram-positive bacteria (*Staphylococcus aureus*, *S. epidermidis*, *Streptococcus mutans*, *Actinomyces naeslundii*, and *Lactobacillus acidophilus*), three Gram-negative bacteria (*Escherichia coli*, *Proteus mirabilis*, *Prevotella intermedia*), and two species of yeast-like fungi (*Candida albicans, C. tropicalis*). All strains were from the collection of the Chair and Department of Medical Microbiology, PUMS. MICs of selected substances were determined by the micro-dilution method using the 96-well plates (Nest Scientific Biotechnology). The methodology was described in our previous publications [123,124]. In wells, we placed Mueller–Hinton broth (Graso, Poland) and the final concentration of microbial inoculums was 10^5^ CFU mL^−1^. The plates were incubated at 35 °C for 24 h. Serial dilutions of each of the substances were performed in the range concentrations of 1.22–2500 μg mL^−1^. The analyses were repeated three times.

### 3.4. Formulation Studies Involving S. baicalensis radix Lyophilized Extract with Chitosan and the Evaluation of Its Quality

#### 3.4.1. Hydrogels Preparation

The compositions of the hydrogel formulations are presented in Table 4. The binary mixture of *S. baicalensis radix* lyophilized extract and chitosan 80:500 was added in two amounts to the placebo samples to achieve its concentration of 2 and 4%. The placebo samples (P1, P2) were prepared in closed 50 mL glass bottles, according to the following procedures. In the case of P1, the deionized water was heated to 90.0 ± 2.0 °C. Then, the desired amounts of sodium alginate and methyl cellulose were added on the surface and mixed for 30 min to obtain clear polymers solution with the use of an RET controlvisc magnetic stirrer (IKA, Staufen, Germany). Subsequently, the mixture was cooled down to 25.0 ± 2.0 °C, and poly(ethylene glycol)—PEG400 was added. After 5 min mixing, poloxamer 407 was poured on the surface and was left for 24 h at 2.0 °C for its entire dissolution. In the case of P2, hydroxypropyl cellulose was added immediately after PEG400 and prior to mixing. The chitosan system with *S. baicalensis radix* extract-loaded formulations was prepared by 15 min mixing of a specific amount of the binary mixture with the placebo samples.

#### 3.4.2. Rheological Experiments

The rheological measurements were carried out with the use of a HAAKE^TM^ RheoStress1 (Thermo Electron Corp., Waltham, MA, USA) rotational rheometer. The rheometer was equipped with a HAAKE^TM^ DC30 thermostat. The titanium plate–plate geometry (35 mm) was used for the measurements with the standard gap of 1.0 mm. After lowering the upper plate, the excess of the sample was gently removed by a spatula to avoid any unwanted shearing. The temperature during measurements was set at 32.0 ± 0.5 °C. The analysis and calculation were performed on HAAKE^TM^ RheoWinTM Data Manager Software (Thermo Electron Corp., Waltham, MA, USA). A fresh sample were used for each measurement. Each assay was conducted in triplicate. The mean values and standard deviations of the obtained parameters were reported.

Steady shear experiments involved flow curves (CR, controlled rate) and a temperature ramp test (TS, temperature sweeping). The flow curves (Figure S2) were plotted as the dependence of the shear stress on the shear rate at 37.0 ± 0.2 °C. The pre-shearing stage included shearing of the samples with a 2.0 1 s^−1^ rate for 5 s. Then, the shear rate increased from 0 to 200.0 s^−1^ over 30 s. The temperature sweeping was performed in the range of 22.0–32.0 ± 0.2 °C under a constant shear rate of 10.0 1 s^−1^. The changes were plotted as the dependence of dynamic viscosity vs. temperature (Figure S3).

Oscillatory shear measurements included stress sweeping (SS) and frequency sweeping (FS) experiments. Stress sweeping was performed with a two-step procedure. At a constant frequency of 1 Hz, the samples were exposed to increasing and decreasing oscillatory stress in the following order 1.0→300.0→1.0 Pa. The oscillatory stress (*τ*), storage modulus (*G*′), and loss modulus (*G*″) values were plotted on a logarithmic sale (Figure 5). For frequency sweeping, the samples were exposed to increasing frequency (f = 1.0–100.0 Hz) at constant oscillatory stress (2.0 Pa). The results are presented in a logarithmic scale as the dependence of *G*′, *G*″ on f (Figure S4).

#### 3.4.3. In Vitro Dissolution Studies

Drug release experiments were performed for hydrogel formulations with the use of vertical Franz cells (PermeGear Hellertown, Pennsylvania, USA), each containing 8 mL of acceptor solution composed (per liter) of potassium chloride (1.20 g), sodium chloride (0.85 g), di-potassium hydrogen orthophosphate (0.35 g), magnesium chloride (0.05 g), calcium chloride (0.20 g), and xylitol (20.0 g) (pH was adjusted to 6.6 by 1 M HCl). The cells were equipped with regenerated cellulose membranes (Visking^®^ dialysis tubing, SERVA Electrophoresis GmbH, Heidelberg, Germany) with a pore diameter ca. 25 Å. The membranes were kept immersed in the acceptor fluid at 37.0 ± 0.5 °C for 24 h before the experiment. The liquid samples (1.0 mL) were placed at the donor compartment and spread evenly on the surface of the artificial membrane. The effective diffusion area of the employed cells was 0.999 cm^2^. The receptor fluid during the test was stirred at 200 rpm and kept at a temperature of 37.0 ± 0.5 °C. The samples (1.0 mL) were taken from the receptor compartment after 30, 60, 120, 180, 240, 300, and 360 min and replaced immediately with an equal volume of receptor fluid. The drug concentration in the collected samples was determined with the validated HPLC method described above.

#### 3.4.4. Mucoadhesive Properties

A TA.XT.Plus Texture Analyzer (Stable Microsystems, Godalming, UK) equipped with the measuring system Rig-G/MUC was used for the mucoadhesion test [125,126].

Porcine cheeks were obtained from the Bost slaughterhouse (Turośń Kościelna, Poland). Tissue excised immediately after animal death was rinsed with isotonic saline solution and frozen at −20 °C. Samples were defrosted at ambient temperature and cut into pieces (with 3 mm thickness) prior mucoadhesion studies. A fragment of epithelium excised from porcine cheek was attached to the thermostated steel plane (37.0 ± 1.0 °C) with cyanoacrylate glue and kept for 5 min prior the experiment. Each gel sample (1 mL) kept at ambient temperature for 24 h before analysis was carefully set on the upper probe with a syringe to prevent air bubbling and secured with the attached support collar. Then, the probe was lowered on the surface of the porcine tissue with a constant speed of 0.5 mm s^−1^ and a contact force of 0.3 N was applied for 60 s. Afterwards, the two surfaces were separated at a constant speed of 0.1 mm s^−1^. The maximum detachment force (*F*_max_) as a function of displacement was recorded from Texture Exponent 32 software, and the work of mucoadhesion (*W*_ad_) expressed in µJ per tissue area was calculated from the area under the force versus distance curve. Cellulose paper was used as a negative control and commercial oromucosal gel based on carbomer with an extract of *S. baicalensis radix* Baikadent^®^ (batch number 030121, expiry date 07.2022, Herbapol, Poland) was applied as a positive control. Each experiment was carried out at least five times [127].

A statistical analysis was carried out with Statistica 12.0 software. The normality of results was checked using the Shapiro–Wilk test. The differences among the mean values of mucoadhesiveness were tested using the Kruskal–Wallis test with post hoc Dunn’s test for multiple comparisons. Differences between groups were considered to be significant at *p* < 0.05.

## 4. Conclusions

In the present studies, thermosensitive semi-solid formulations containing the binary mixture of the *S. baicalensis radix* lyophilized extract and chitosan were developed for periodontitis treatment. The combination of *S. baicalensis radix* extract and chitosan exhibited synergistic effects toward ferrous ion chelating activity, inhibition of hyaluronidase, and pathogen growth. For this purpose, temperature-sensitive poloxamer 407 and sodium alginate as an ion-sensitive polymer, stiffening the structure in contact with ions present in saliva, were used. In addition, two types of polymers were added to more efficiently bind the preparations to the site of administration and thus extend the residence time. As a result, the developed innovative gels showed a tendency to have a liquid consistency at 25–27 °C, while they thickened immediately after exceeding it. Therefore, it will be possible to inject the preparations into the periodontal pocket and fill it thoroughly, which is an unquestionable advantage over commercial gels available on the market. After thickening, the gels showed a prevalence of elastic properties over viscous ones, while maintaining the shear thinning behavior. The presence of chitosan in a binary mixture improved the binding strength of the formulations with the mucosa significantly.

## Figures and Tables

**Table 1:** Apparent permeability coefficients (P*_app_*) values of standards, *S. baicalensis radix* extract, and binary mixtures.

	P*_app_*(× 10^−6^ cm s^−1^)		
	Baicalin	Baicalein	Wogonin
Standard	0.02 ± 0.01	91.56 ± 2.72	57.23 ± 5.42
SBE	n.d.	116.58 ± 3.97	134.43 ± 3.59
SBE/Cs 80:500	n.d.	119.05 ± 7.64	133.66 ± 6.44
SBE/Cs 80:1000	n.d.	129.03 ± 1.34	130.60 ± 3.58

Compounds with P*_app_* < 0.1 × 10^−6^ cm s^−1^ = low-permeable compound, those with P*_app_* ≥ 1 × 10^−6^ cm s^−1^ = high-permeable compounds indicated values are means (±SD, *n* = 3); n.d.—not detected in the acceptor.

**Table 2:** Antimicrobial activity of standards, *S. baicalensis radix* extract, and binary mixtures.

Pathogen	MIC (µg mL^−1^)						
	SBE	SBE/Cs 80:500	SBE/Cs 80:1000	Cs 80:500	Cs 80:1000	Baicalin	Baicalein
*A. naeslundii*	1250	>2500	>2500	1250	1250	312.5	156.25
*L. acidophilus*	625	39.1 *	>2500	78.1	156.25	156.25	312.5
*S. aureus*	1250	78.125 *	2500	156.25	2500	625	625
*S. epidermidis*	1250	78.125 *	>2500	156.25	>2500	625	625
*S. mutans* Clarke ATCC 25175	>2500	156.25 *	>2500	78.1	156.25	625	312.5
*E. coli*	1250	312.5 *	2500	625	2500	1250	312.5
*P. mirabilis*	1250	2500	2500	2500	2500	1250	1250
*P. intermedia* ATCC 25611	625	1250	>2500	1250	1250	1250	625
*C. albicans*	1250	156.25 *	2500	312.5	2500	625	1250
*C. tropicalis*	1250	156.25 *	2500	312.5	2500	1250	625

* decrease the MIC value compared with MIC of *S. baicalensis radix* extract.

**Table 3:** Rheological parameters calculated for placebo and SBE/Cs 80:500 loaded hydrogels.

	P1	P2	F1-2	F1-4	F2-2	F2-4
	**Controlled rate**					
K	188.53 ± 5.42	9.07 ± 0.64	186.67 ± 4.00	113.17 ± 7.06	---	---
*n*	0.11 ± 0.00	0.74 ± 0.02	0.10 ± 0.00	0.11 ± 0.00	---	---
τ_0_	---	316.53 ± 8.46	---	---	---	---
	**Temperature sweeping**					
T_sol–gel_	26.65 ± 0.11	25.68 ± 0.02	26.75 ± 0.04	26.28 ± 0.03	26.62 ± 0.02	25.80 ± 0.02
η_sol–gel_	8.45 ± 0.07	13.02 ± 0.47	12.21 ± 0.04	9.62 ± 0.03	10.61 ± 0.05	16.93 ± 0.03
	**Stress sweeping**					
LVR	74.83 ± 9.05	106.34 ± 10.01	84.48 ± 20.90	66.31 ± 18.08	62.07 ± 10.13	70.63 ± 13.13
Cross-over ↑	158.43 ± 1.40	189.87 ± 5.09	165.97 ± 13.90	147.82 ± 20.64	168.77 ± 11.12	195.77 ± 10.37
Cross-over ↓	92.88 ± 3.13	122.67 ± 12.19	92.73 ± 0.35	89.10 ± 5.15	87.73 ± 4.10	122.10 ± 7.24

K—consistency coefficient, *n*—power law index, τ0—yield point, Tsol–gel—gelation temperature, ηsol–gel—dynamic viscosity at Tsol–gel, LVR—linear viscoelastic region, Cross-over ↑—G′ = G″ point at increasing stress ramp, Cross-over ↓—G′ = G″ point at decreasing stress ramp.

**Table 4:** Percent composition of the prepared formulations.

Component	AlgNa	MC	HPC	PEG400	P407	DW	SBE/Cs 80:500
P1	0.4	0.6	---	2.0	17.0	80.0	---
P2	0.4	---	0.6	2.0	17.0	80.0	---
F1-2	P1 98.0						2.0
F1-4	P1 96.0						4.0
F2-2	P2 98.0						2.0
F2-4	P2 96.0						4.0

## Data Availability

Data are contained within the article and Supplementary Materials.

## Supplementary Materials

The following are available online at https://www.mdpi.com/article/10.3390/ijms222111319/s1.
