# Supplementary material for: Development and Evaluation of Thermosensitive Hydrogels with Binary Mixture of Scutellariae baicalensis radix Extract and Chitosan for Periodontal Diseases Treatment
Source: Int J Mol Sci. 2021 Oct 20;22(21):11319. doi: 10.3390/ijms222111319 (PMC8583119; doi:10.3390/ijms222111319)
Supplement: Supplementary file 1 [file ijms-22-11319-s001.zip › ijms-1433034-supplementary.pdf]

Supplementary material

Table S1. Validation parameters of the flavones determination by the UHPLC-DAD method.

Parameter	**Baicalin**	**Baicalein**	**Wogonin**
Linearity: y = ax + b	**y = 0.2536x – 0.7974**	**y = 0.472 – 2.6637**	**y = 0.3408x**
a ± S_a	0.2536 ± 0.009	0.472 ± 0.0036	0.3408 ± 0.0017
b ± S_b	-0.7974 ± 0.1383	-2.6637 ± 0.5401	insignificant (α = 0.05)
correlation coefficient (r)	1.0000	0.9999	0.9999
Limit of detection (LOD): LOD = 3 SD/a (μg mL^{-1})	2.42	5.07	3.70
Limit of quantification (LOQ): LOQ = 10 SD/a (μg mL^{-1})	7.32	15.36	11.22
Range of linearity (μg mL^{-1})	10–250	10–250	10–250
Precision, RSD			
50 [μg mL^{-1}]	0.9092	0.4488	0.7388
100 [μg mL^{-1}]	1.4755	1.3150	0.8626
150 [μg mL^{-1}]	1.2758	1.1209	0.5473
Intra-day, RSD			
50 [μg mL^{-1}]	1.9967	1.7808	1.4532
100 [μg mL^{-1}]	1.2791	1.9548	2.4567
150 [μg mL^{-1}]	1.1350	1.1504	3.6224
Retention time (min)	30.95	47.36	60.41

Sa standard deviation of slope; *Sb* standard deviation of intercept, t calculated values of the Student's t test, $t_{\alpha,f}$ = 2.571 critical values of the Student's test for degrees of freedom f = 5 and significance level α = 0.05.

Table S2. The drug flux and the average cumulative amount per area during dissolution studies of formulations with binary mixture of *S. baicalensis radix* lyophilized extract and chitosan.

	**Drug Flux (*Jss*) [μg $cm^{-2}h^{-1}$]**	**b**	**Correlation Coefficient (r)**	**Release Coefficient (k_r) [cm h^{-1}]**	**Average Cumulative Amount Per Area at 6 h (Q_{6h}) [μg cm^{-1}]**
			**Baicalin**		
**F1-2**	8.29 ± 0.14	22.33 ± 0.36	0.999 ± 0.000	2.37 ± 0.01	71.69 ± 1.30
**F1-4**	11.55 ± 0.39	20.31 ± 0.24	0.999 ± 0.000	1.62 ± 0.05	89.38 ± 2.00
**F2-2**	6.44 ± 0.51	22.81 ± 0.30	0.995 ± 0.004	1.78 ± 0.19	60.75 ± 2.99
**F2-4**	8.74 ± 1.88	19.15 ± 0.62	0.985 ± 0.018	1.23 ± 0.26	72.64 ± 10.45
			**Baicalein**		
**F1-2**	7.09 ± 0.10	43.69 ± 0.07	0.995 ± 0.001	5.62 ± 0.08	85.62 ± 0.64
**F1-4**	7.95 ± 0.15	43.56 ± 0.09	0.996 ± 0.000	3.16 ± 0.06	90.65 ± 0.86
**F2-2**	7.07 ± 0.04	43.75 ± 0.04	0.995 ± 0.000	5.61 ± 0.03	85.63 ± 0.25
**F2-4**	7.75 ± 0.10	43.59 ± 0.07	0.999 ± 0.000	3.07 ± 0.04	89.60 ± 0.47
			**Wogonin**		
**F1-2**	0.09 ± 0.01	-0.06 ± 0.01	0.981 ± 0.007	0.17 ± 0.02	0.44 ± 0.06
**F1-4**	0.17 ± 0.08	-0.13 ± 0.08	0.985 ± 0.002	0.17 ± 0.08	0.88 ± 0.44
**F2-2**	0.11 ± 0.00	-0.09 ± 0.00	0.975 ± 0.006	0.21 ± 0.00	0.59 ± 0.02
**F2-4**	0.19 ± 0.03	-0.10 ± 0.05	0.963 ± 0.010	0.19 ± 0.03	1.10 ± 0.14

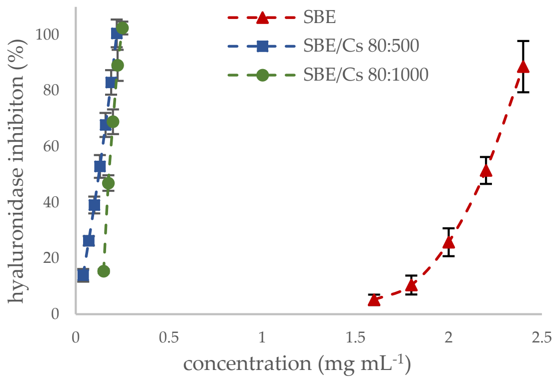

Figure S1. The plot presenting inhibition activity of *S. baicalensis radix* extract and binary mixtures toward hyaluronidase enzyme (mean ± S.D., *n* = 6).

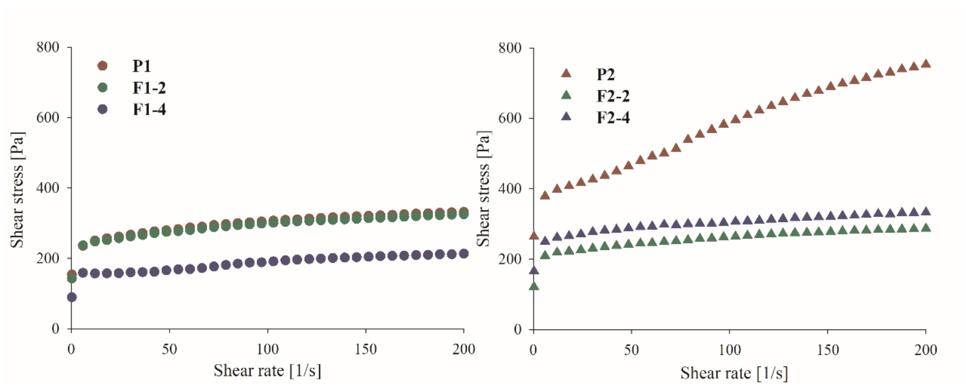

(**a**) (**b**)

Figure S2. The flow curves of (**a**) the gel samples without and (**b**) after addition of the SBE/Cs 80:500.

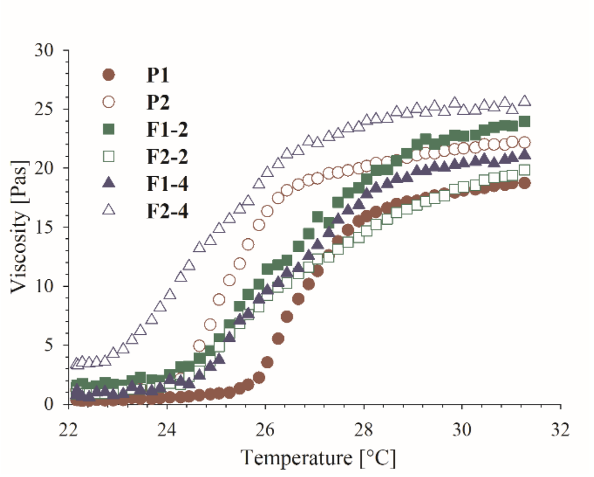

Figure S3. The temperature sweeping of the gel samples.

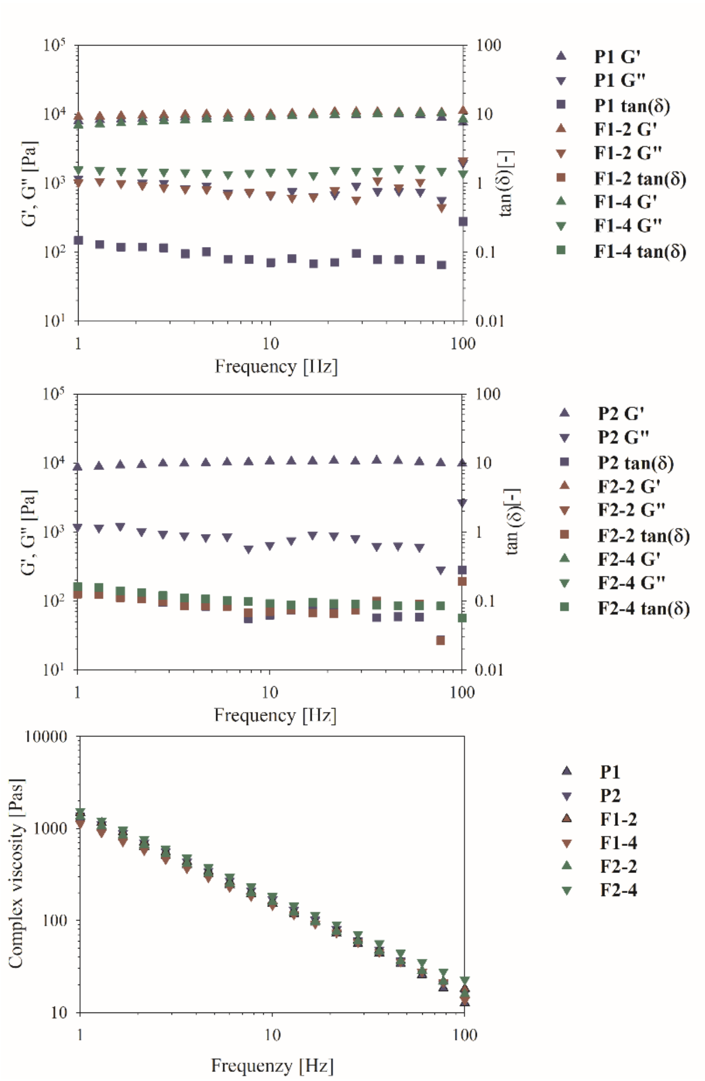

Figure S4. The oscillatory frequency sweeping of the gel samples.
